# A post-COVID syndrome curriculum for continuing medical education (CME): in-person versus livestream

**DOI:** 10.3389/fmed.2024.1438068

**Published:** 2024-08-27

**Authors:** Michael Mueller, Ravindra Ganesh, Darrell Schroeder, Thomas J. Beckman

**Affiliations:** Mayo Clinic, Rochester, MN, United States

**Keywords:** flipped classroom, post-COVID “long-haulers,” in person learning, livestream learning, continuing medical education (CME)

## Abstract

**Background:**

Nearly 30% of patients with COVID-19 infection develop post-COVID Syndrome. Knowledge of post-COVID Syndrome is evolving, creating the need for adaptable curricula. Flipped classrooms (FC) are flexible and dynamic with demonstrated utility in continuing medical education (CME), yet there has been no research on application of FCs, or comparisons between livestream and in-person learning, in post-COVID CME.

**Methods:**

We implemented a novel post-COVID curriculum using FCs for in-person and livestream participants at four Mayo Clinic CME conferences. Outcomes were validated measures of knowledge; perceptions of FCs and CME teacher effectiveness; and learner engagement. Pre-conferences surveys were a post-COVID knowledge test and the Flipped Classroom Perception Inventory (FCPI). Post-conference surveys were a post-COVID knowledge test, the FCPI, the CME Teaching Effectiveness Instrument (CMETE), and the Learner Engagement Inventory (LEI). Pre-post knowledge and FCPI scores were analyzed using linear mixed models. CMETE and LEI were compared for in-person versus livestream participants using the Wilcoxon rank-sum test.

**Results:**

Overall, 59 participants completed the pre-test, and 72 participants completed the post-test, surveys. Participants were predominantly female (58%), were in nonacademic group practices (65%), and lacked prior experience with flipped classrooms (83%). Following the presentations, participants showed significant improvements in post-COVID knowledge (47% correct precourse to 54% correct postcourse, *p*-value = 0.004), and a trend toward improved FCPI scores. Teaching effectiveness, learner engagement, and pre-post change in COVID knowledge did not differ significantly between participants of in-person versus livestream sessions.

**Conclusion:**

This post-COVID FC curriculum was feasible and associated with improved knowledge scores among a diverse population of physician learners in CME, without any apparent compromise in learner engagement, or in perceptions of teaching effectiveness and FCs, among livestream versus in-person participants.

## Background

Amidst the COVID pandemic there is an increased need for novel methods to disseminate rapidly evolving medical information effectively and safely (1–3). Post-COVID syndrome (PoCoS) is a constellation of symptoms persisting greater than three months after initial onset of acute COVID symptoms (4). PoCoS is characterized by fatigue, orthostatic intolerance, or diffuse pain disproportionate to objectively measured markers of tissue damage. In addition to being significantly function limiting, PoCoS is common, affecting an estimated 10–30% of patients with COVID (5, 6). Barriers to disseminating information on PoCoS include lack of standardized information, rapidly evolving data and treatment recommendations, and limited existing curricula.

There is growing interest in flipped classroom (FC) models as a flexible, dynamic, and accessible teaching modality to disseminate information safely, effectively, and rapidly during in the setting of the COVID pandemic. As opposed to traditional models, FC models require that course attendees prepare before class participation and then use face-to-face learning time to apply key concepts. FC models incorporate higher order Bloom’s taxonomy functions such as application, analysis, and evaluation in the face-to-face settings, which enhances opportunities for feedback and knowledge assessments (7–10).

Previously, FC models have been successfully studied and implemented at the Mayo Clinic through a QI curriculum for residents and in a CME setting primarily involving academic faculty members (11, 12). To our knowledge, utilization of FC methodologies in the setting of PoCoS curricula has not been studied.

## Project design/methods

Setting: We conducted a cross-sectional survey with pre/post-test analyses of all participants at the post-COVID talks at the following Mayo Clinic CME events: The Practice of Internal Medicine Conference (POIM) on 3 May 2022, the Medically Unexplained Symptoms Conference on 18 August 2022, the Updates in Internal Medicine Conference on 22 October 2022, and the Medically Unexplained Symptoms Conference on 10 August 2023. These courses are all accredited by the Mayo School of Continuous Professional Development, range in attendance from 60 to 220 attendees, and qualify for between 19.5 and 26.25 h of CME credit. Each course consisted of 30 to 60-min podium presentations and small-group breakout sessions, and all courses were offered in both in-person and livestream formats. This study was deemed exempt by the Mayo Clinic Institutional Review Board (ID 21-012865).

Study variables: Demographic variables of course participants, collected on the pre-survey, included age (years: 20–30, 31–40, 41–50, 51–60, ≥ 61), sex (M/F), practice setting (academic, group, training program, other), specialty (internal medicine, family medicine, medical specialty, non-medical specialty), practice location (northeast, southeast, midwest, southwest, west, international), pre-FC module completion (< 50%, ≥ 50%), and prior FC experience (Y/N). The outcome variables were a 10-item multiple choice post-COVID knowledge questionnaire, the 8-item Flipped Classroom Perception Inventory (FCPI), the 8-item CME Teaching Effectiveness Instrument, and the 8-item Learner Engagement Inventory (11, 13–15). Please see Supplementary Addendums 1–4 for the actual instruments.

Before the conference, participants received the multiple choice post-COVID knowledge questionnaire which was reviewed, revised, and updated for accuracy prior to each CME course. In addition, participants received the Flipped Classroom Perception Inventory (FCPI), which was designed to measure baseline knowledge and perceptions of flipped classroom curricula. After the conference, participants again received the multiple choice questionnaire. They also received the FCPI, the CME Teaching Effectiveness instrument, and Learner Engagement Inventory, all of which have been validated and published by Mayo authors (11, 13).

Study design: This was a two-group group, pre/post-test comparison study of a post-COVID curriculum using FCs among attendees at four Mayo Clinic CME courses.

Study intervention: Before the conference, participants were given access to online didactic materials reviewing key concepts to understanding post-COVID syndrome (see Supplementary Addendum 5 for a copy of the didactic material used). Modules were developed, reviewed, and revised by experts who designed and staff the Mayo Clinic Post COVID Care Clinic, and they were updated for accuracy prior to each CME course. The in-person conference presentation for post-COVID syndrome consisted of case-based presentations by the experts listed above, followed by a 30-s “think, pair, share” discussion among conference participants. Livestream participants were given a 30-s pause for reflection prior to proceeding to the next case.

Data analysis: Categorical data are presented as numbers and percentages. Continuous data are summarized as mean and SD. Knowledge and FCPI scores were analyzed using linear mixed models with time (pre vs. post) as the explanatory variable of interest and participant included as a random effect to account for the repeated measures design. These analyses were performed using all available data and also using data only for those who completed both the pre- and post-surveys. In order to assess whether changes from pre to post differed between those who attended in person versus livestream, a secondary analysis was performed which included mode of attendance (in person vs. livestream) and the mode of attendance—by—time interaction. CMETE and LEI were available for those who completed the post-survey and were compared for in-person versus livestream participants using the Wilcoxon rank-sum test. Statistical Significance was set at α < 0.05. Statistical analyses were conducted with SAS version 9.4 software (SAS Institute Inc., Cary, NC, USA).

## Results

The pre-survey was completed by 59 participants and the post-survey was completed by 72 participants; with 26 participants completing both surveys. Demographics of those who completed the pre-survey are presented in Table 1. The majority of participants were from a group practice (65%), and most reported their specialty as internal medicine generalist (42%) or family medicine (38%).

**TABLE 1:** Respondent characteristics.

Characteristic	All pre-survey respondents (*N* = 59*)	Those who responded to both pre- and post-surveys (*N* = 26*)
**Gender**		
Female	32 (58%)	11 (46%)
Male	23 (42%)	13 (54%)
**Age, years**		
20 to 30	1 (2%)	1 (4%)
31 to 40	16 (30%)	2 (8%)
41 to 50	11 (20%)	6 (25%)
51 to 60	12 (22%)	5 (21%)
61 or greater	14 (26%)	10 (42%)
**Practice setting**		
Academic practice	12 (22%)	5 (21%)
Group practice	36 (65%)	13 (54%)
Training program	2 (4%)	2 (8%)
Other	5 (9%)	4 (17%)
**Specialty**		
Internal medicine—generalist	23 (42%)	12 (50%)
Internal medicine—subspecialist	4 (7%)	2 (8%)
Family medicine	21 (38%)	6 (25%)
Non-internal medicine	1 (2%)	1 (4%)
I do not practice medicine	6 (11%)	3 (13%)
**Location**		
US Northeast	3 (5%)	1 (4%)
US Southeast	6 (11%)	1 (4%)
US Midwest	24 (44%)	11 (46%)
US Southwest	8 (15%)	4 (17%)
US West	13 (24%)	6 (25%)
Outside the US	1 (2%)	1 (4%)
**How much of the precourse material did you review?**		
< 50%	38 (70%)	15 (63%)
> 50%	16 (30%)	9 (38%)
**Have you had experience with prior flipped classroom**		
**curricula?**		
No	45 (83%)	23 (96%)
Yes	9 (17%)	1 (4%)

*Due to missing data the sum of the number of respondents across categories for a given characteristic may be less than the total N.

Results of the analyses of the FCPI and knowledge scores are summarized in Table 2. From the analysis which included all available data, the mean knowledge score increased significantly from pre to post (mean change = 0.8, 95% CI 0.3 to 1.3, *p* = 0.004), and the FCPI score was also observed to increase (mean change = 0.2, 95% CI 0.0 to 0.3, *p* = 0.053). Similar results were obtained from the analysis which was restricted to participants who completed both the pre- and post-surveys. From supplemental analysis, the changes from pre to post were not found to differ significantly between those who attended in person versus livestream (interaction *p* = 0.932 and *p* = 0.458 for knowledge and FCPI, respectively). For participants who completed the post-survey the CMETE and LEI scores are summarized in Table 3. For these outcomes, there were no significant differences found between those who attended in person versus livestream (*P* = 0.967 for CMETE score, *p* = 0.805 for LEI score).

**TABLE 2:** FCPI and knowledge scores from pre-survey and post-survey.

	All responses				Participants who responded to both pre-survey and post-survey			
**Characteristic**	**Pre (*N* = 59^†^)**	**Post (*N* = 72^†^)**	Delta (95% CI)*	*P* *	**Pre (*N* = 26)**	**Post (*N* = 26)**	Delta (95% CI)*	*P* *
FCPI score	3.7 ± 0.5	3.8 ± 0.8	0.2 (0.0, 0.3)	0.053	3.7 ± 0.6	3.9 ± 0.7	0.2 (0.0, 0.4)	0.058
Knowledge score	4.7 ± 1.5	5.4 ± 1.7	0.8 (0.3, 1.3)	0.004	4.8 ± 1.2	5.9 ± 1.4	1.0 (0.3, 1.7)	0.006

*Data were analyzed using a linear mixed model with time (pre vs. post) as the explanatory variable, and participant included as a random effect to account for repeated measurements. Results are summarized by presenting the estimate and 95% confidence interval for the difference between time periods (post—pre).

^†^ participant had missing data for FCPI in the pre-survey and one participant had missing data for the knowledge questionnaire in the post-survey.

**TABLE 3:** CMETE and LEI scores from post-survey: Overall and according to mode of attendance.

Characteristic	Overall (*N* = 72^†^)	Livestream (*N* = 30)	In person (*N* = 42^†^)	*P* *
CMETE score				0.967
Mean ± SD	4.4 ± 0.8	4.4 ± 0.7	4.4 ± 0.9	
Median (25th, 75th)	4.5 (4.1, 5.0)	4.4 (4.3, 4.9)	4.6 (4.0, 5.0)	
LEI score				0.805
Mean ± SD	4.3 ± 0.8	4.4 ± 0.7	4.3 ± 0.9	
Median (25th, 75th)	4.5 (4.0, 4.9)	4.6 (4.1, 4.9)	4.5 (4.0, 4.9)	

*Wilcoxon rank sum test.

^†^ participant had missing data for CMETE.

## Discussion

To our knowledge, this is the first study to demonstrate the feasibility and effectiveness of a post-COVID FC curriculum among postgraduate clinicians. The FC curriculum was associated with improved knowledge scores for both in person and livestream attendees. Furthermore, teaching effectiveness scores were not significantly different for in person versus livestream attendees, suggesting that this post-COVID FC curriculum is equally effective for distance learners.

Approximately 10 to 30% of patients with COVID-19 develop post-COVID Syndrome, which results in 2 to 4 million unemployed and $170 billion in lost wages annually in the United States (16–18). Few post-COVID curricula exist, and developing these curricula is challenging due to lack of standardization and rapidly evolving treatment recommendations (16), which highlights a need for post-COVID curricula that are effective, adaptable, and feasible.

We are unaware of previous curricula—let alone with the incorporation of FC methodology—for the management of post-COVID Syndrome. This curriculum was associated with similar knowledge gains for both in person and livestream models, and for attendees at multiple CME courses over 16 months, which speaks to the curriculum’s adaptability. Prior experience with FC models did not impact on knowledge gains. Finally, there was nonsignificant trend toward pre-post course perceptions of the FC approach, which is consistent with prior FC research (12).

Studies have suggested that learner engagement and teaching effectiveness are strongly correlated (13), and that in-person flipped classroom sessions are generally favored over online sessions (11, 12). However, this study did not reveal significant differences between in person versus livestream attendees with respect to teaching effectiveness or learner engagement scores, which indicates that, at least for this post-COVID FC curriculum, livestream attendance may be just as feasible and effective as the more resource intensive, in person sessions. Moreover, CME Teaching Effectiveness scores for this curriculum compared favorably with what was previously reported in the literature (15).

This study has several limitations. According to some traditional models, flipped classroom teachers interact with their students to check in, confirm understanding, and/or correct mistakes. A limitation of this study is that the instructors were teaching large audiences from the podium within strict time constraints, which precluded any informal interaction with the learners. Decreased learner engagement may have hampered the effectiveness of the extensive, precourse, FC learning materials. Future research should endeavor to improve learner engagement and satisfaction by implementing more streamlined course materials, and by offering greater incentives (19, 20). Participants were primarily older and practiced in nonacademic settings, which may limit the generalizability of flipped classroom data to other populations. The study relied on self-reporting of precourse material review, which could not be verified objectively. Moreover, although there were significant improvements in knowledge scores following the curriculum intervention, the absolute percentages correct on the knowledge assessments were not very high. Another potential explanation for the low knowledge scores is that that post-COVID syndrome is a very new and recent disorder; therefore, there remains little experience with this topic among general clinicians, and the pathophysiological mechanisms are still being determined. Finally, the surveys had generally low response rates.

In summary, we present the results of research on a novel post-COVID FC curriculum. The curriculum intervention was associated with increased pre-post knowledge scores, and with CME Teaching Effectiveness scores that compared favorably with prior CME research. Validated outcome measures for knowledge acquisition, teaching effectiveness, and learner engagement were similar for in person and livestream participants, suggesting that distance learning, which is less resource intensive, may be a feasible option for content delivery. We anticipate that future endeavors to streamline the precouse FC material will lead to further enhancements in knowledge acquisition and learner engagement.

## Data Availability

The raw data supporting the conclusions of this article will be made available by the authors, without undue reservation.
