# Supplementary material for: A post-COVID syndrome curriculum for continuing medical education (CME): in-person versus livestream
Source: Front Med (Lausanne). 2024 Aug 27;11:1438068. doi: 10.3389/fmed.2024.1438068 (PMC11385658; doi:10.3389/fmed.2024.1438068)
Supplement: Supplementary file 1 [file Presentation_1.pdf]

Addendum 1: Post-COVID Syndrome Knowledge Assessment (MCQs)

1. A 50-year old man had COVID-19 infection 6 weeks ago. He was hospitalized for 6 days and received remdesivir and dexamethasone and continues to have persistent dyspnea. What is the next best evaluation?

a. Chest X-ray

b. Non-contrast chest CT

c. Pulmonary function testing

d. Chest CT pulmonary angiogram

e. Echocardiogram

2. Which of the following factors would NOT be considered a risk factor for post-COVID pulmonary fibrosis in a COVID-positive patient?

a. Gender

b. Smoking history

c. Intubation during acute COVID infection

d. CRP of 263 mg/l

e. Persistent dyspnea after COVID infection

3. A 62 year old man with a history of hypertension was admitted with severe COVID 19 pneumonia. He was treated with remdesivir and dexamethasone. He is now 6 weeks after his initial diagnosis. He continues to have shortness of breath and cough. Chest CT shows persistent, bilateral ground glass opacities. He denies fevers. White blood count is normal. What would be the next best step in management?

a. Pulmonary function testing

b. Prednisone

c. Bronchoscopy

d. Echocardiogram

e. Remdesivir

4. You are seeing a 43-year old female with a history of asthma has persistent dyspnea after COVID-19 infection. CT chest, echocardiogram, and pulmonary function testing are normal, and she is already

engaged in a pulmonary rehabilitation program. Which of these next interventions would be most appropriate?

a. Hyperbaric oxygen therapy

b. Nintedanib

c. Furosemide

d. Inhaled fluticasone/vilanterol

e. Nebulized morphine

5. Which of the following post-COVID syndrome phenotypes is most common?

a. Fatigue

b. Orthostasis

c. Myalgia

d. Headache

e. Chest pain

6. Which of the following characteristics is NOT a risk factor for development of post-COVID syndrome?

a. Female gender

b. Hospitalization during acute COVID-19 infection

c. Mechanical ventilation during acute COVID-19 infection

d. Prior medical history of fibromyalgia

e. Ashkenazi Jewish descent

7. You are seeing a patient with severe orthostatic tachycardia 4 months after acute COVID-19 infection. Symptoms include tachycardia, involuntary tremors, night sweats, and peripheral skin discoloration, and a norepinephrine level obtained while standing was 1100 pg/ml (expected < 900). Which of the following treatments would be most appropriate?

a. Prednisone

b. Propranolol

c. Fludrocortisone

d. Midodrine

e. Ivabradine

8. Which of the following laboratory or clinical findings would be most likely found in a patient with post-COVID syndrome?

a. ESR of 82 mm/hr

b. WBC of 2700 / microliter

c. IL-6 of 4.1 pg/ml

d. MRI brain showing chronic-appearing small vessel ischemia

e. Nocturnal pulse oximetry concerning for severe untreated obstructive sleep apnea

9. Which of the following interventions would be least appropriate for a patient with persistent parosmias after COVID-19 infection?

a. Smell retraining programs

b. Transcranial magnetic stimulation

c. Budesonide sinus irrigations

d. Omega-3 fatty acid supplementation

10. In the right clinical context, which of the following serology results would be most concerning for persistent COVID-19 infection?

a. COVID-19 nucleocapsid antibody positive, COVID-19 PCR swab negative

b. COVID-19 nucleocapsid antibody positive, COVID-19 PCR swab positive

c. COVID-19 nucleocapsid antibody negative, COVID-19 PCR swab positive

d. COVID-19 nucleocapsid antibody negative, COVID-19 PCR swab negative

e. COVID-19 spike antibody positive, COVID-19 PCR swab positive

Addendum 2: Flipped Classroom Perceptions Inventory (FCPI).

1. Interactive, applied, in-class activities enhance my learning.
2. I participated and engaged in activities during the post-COVID course.
3. In-class application of core content enhances my learning.
4. In-class discussions of core content enhance my learning.

5. Working in a group enhances my learning.
6. Online modules greatly enhance my learning.
7. Learning key content before the session enhances my learning.
8. The combination of online modules with in-class application improves my learning.

Addendum 3: CME Teaching Effectiveness Instrument (CMETE)

1. The speakers presented information in a clear and organized manner.
2. Content was relevant.
3. Examples or cases were given to facilitate my understanding.
4. The slides added to the effectiveness of the presentation.
5. The speakers included opportunities to learn interactively.
6. The speakers provided adequate evidence to support the content, and presented neither too much nor too little.
7. An appropriate amount of information was provided – neither too much nor too little.
8. The speakers summarized key points.

Addendum 4: Learner Engagement Inventory (LEI)

1. I enjoyed this presentation.
2. I was interested in this presentation.
3. I participated in this presentation.
4. I avoided distractions.
5. I was an active learner.
6. I was absorbed in this presentation.
7. I will apply this presentation to my practice.
8. I am motivated to learn more about this topic.

Addendum 5: Sample precourse didactic materials

Definition:

- Definitions are terms are highly variable (PASC, post-COVID syndrome, post-COVID conditions) – our consensus clinical definition is listed above
- Believed to be a **nociplastic** process as opposed to a nociceptive or neuropathic process
  - Nociplastic: Pain/symptoms generated by changes in brain processing and/or signaling
  - Nociceptive: Pain/symptoms generated by direct or threatened damage to peripheral tissues
  - Neuropathic: Pain/symptoms generated by direct or threatened damage to nerves
- Symptoms are **heterogeneous** and may be present in the absence of extensive routine workups for nociceptive drivers (examples: inflammatory markers, imaging, EMG, CMP, CBC, thyroid function testing, adrenal function testing)
- Risk factors: Female predominant, increased risk with severe COVID requiring hospitalization
  - Nociplastic syndromes such as fibromyalgia, ME/CFS may increase risk of long COVID

How should I work up long COVID?

- Currently, there is **no testing** that can confirm a diagnosis of long COVID
  - Experimental testing, such as cytokine profiles (especially interleukin-6) and brain PET CT may show abnormalities
- Testing strategy should be based on pretest odds of disease, but is often more extensive due to what little is known about long COVID
- Strategy is to **rule out nociceptive contributors to long COVID symptoms**
- **Routine testing:** Consider CMP, CBC, D-dimer, thyroid function testing, ESR, CRP
- **Fatigue/Brain fog:** DHEA-sulfate (or adrenal insufficiency testing), ferritin, vitamin B12, vitamin D, overnight oximetry or polysomnography
- **Orthostatic intolerance:** Consider TTE, Holter monitor with symptom diary, and tilt table testing (if available)
- **Widespread pain:** Consider EMG, creatine kinase, ANA, CCP antibodies, rheumatoid factor
- **Dyspnea:** Consider PFTs with methacholine challenge, CT chest (with IV contrast if concern for PE), ambulatory pulse oximetry
- **Persistent anosmia/parosmia:** Consider CT sinuses, ENT referral for laryngoscopy

How should I talk with my patients about long COVID?

- **Reassurance** – post-COVID symptoms are **common** and function-limiting

- Emphasize **nociplastic** nature – while symptoms may be significant, they occur in the absence of objectively measured peripheral tissue damage
- Emphasize the importance of supportive/nonpharmacologic therapy
    - Although some medications are helpful in controlling post-COVID symptoms, no FDA-approved treatments exist for long COVID at this time

What options do I have for treatment of long COVID?

- **Nonpharmacologic therapies: Foundation** of treatment, offer to everyone
    - Biofeedback: Consider MUSE headband if available
    - Mindfulness therapies / optimizing sleep hygiene
    - Physical Therapy/Occupational Therapy: Focus should be on pacing, avoiding energy crashes, and optimizing movement while limiting pain
    - Speech Language Pathology: Helpful for brain fog, inattention, memory, and word-finding difficulties
    - Acupuncture – can be considered in some cases
- Pharmacologic therapies: Focus on primary symptom
    - All therapies are off-label – there are no mandatory prescriptions for long COVID. Scripts are at physician discretion!
    - Make sure to check dosing and medication interactions prior to prescription!

**Medication:**	**Cost:**	**Best for:**	**Dosing strategy:**
L-arginine + Vitamin C	$	Fatigue, brain fog	2000 mg L-arginine BID + 500 mg vitamin C BID
Guanfacine	$	Fatigue, brain fog	1 mg daily
Low-dose naltrexone	$$	Fatigue	4.5 mg daily
Amitriptyline	$	Pain	Start at 25 mg at bedtime, titrate to effect
Propranolol	$	Orthostatic intolerance	Start at 10 mg daily, titrate to effect

Duloxetine	$$	Pain	Start at 20 mg daily, titrate to effect (target dose 60 mg daily)
Pregabalin	$$	Pain	Start at 25 mg daily, titrate to effect
Gabapentin	$	Pain	Start at 300 mg at bedtime, then titrate to effect

Cost key:

$: < $10/month

$$: between $10-$50/month

**Medication:**	**Potential mechanism:**
L-arginine + Vitamin C	May decrease endothelial dysfunction, add antioxidant effects
Guanfacine	Decreases peripheral sympathetic tone, strengthens regulation of attention/behavior in the prefrontal cortex
Low-dose naltrexone	Anti-inflammatory effects on microglial cells
Amitriptyline	Increase concentrations of norepinephrine, serotonin in the synaptic cleft, anticholinergic effects, antimuscarinic effects
Propranolol	Decrease sympathetic tone and heart rate
Duloxetine	Increase concentrations of norepinephrine, serotonin in the synaptic cleft
Pregabalin	GABA agonist, mechanism unknown but increases serotonin levels and decreases excitatory neurotransmitters
Gabapentin	GABA agonist, mechanism unknown but increases serotonin levels and decreases excitatory neurotransmitters

References available upon request – e-mail mueller.michael@mayo.edu for references for the above!

Key reference:

Michael R. Mueller, Ravindra Ganesh, Ryan T. Hurt, Thomas J. Beckman, Post-COVID Conditions, Mayo Clinic Proceedings, Volume 98, Issue 7, 2023, Pages 1071-1078, ISSN 0025-6196, https://doi.org/10.1016/j.mayocp.2023.04.007.
(https://www.sciencedirect.com/science/article/pii/S0025619623001763)
